# The Recent Meeting T. S. M. A.

**Published:** 1891-05

**Authors:** 


### THE RECENT MEETING OF THE STATE MEDICAL ASSOCIATION.

The 28th annual session of this splendid organization has passed into history. It was a fine gathering of men, about four hundred in all; men, in point of dignity and personal appearance, above the average. It was a distinctly representative body of the more advanced element of Texas' practitioners. That the doctors are prospering is evident by larger attendance, better looks and being better dressed than in the past.

It has been our observation through life, that when a man begins to do well in the world, he generally *fattens up*. Some are like Pharoah's cattle, however, the "lean kine (d)" and cannot fatten; but these, when the screws are loosened, and the pressure of the hard times lets up; when they begin to ease up financially, relax their brows, loose that *distrait*, care-worn appearance, dress up, become more companionable—some even jolly, and "prosperity" is visible in every lineament and every act. Judging from appearances most of the delegates at Waco have been successful in a business point of view.

The sessions were well attended and full of interest. Some good papers were read and some indifferent.

A stenographer had been employed to take down discussions, and, as both papers and talks will shortly appear in cold type, readers must judge for themselves; we will make no invidious distinctions.

All went smoothly, on the surface; but, it seems to us, that there never has been, at any previous meeting, so much "log rolling," so much lobbying, so many and such large hall and stair-way caucusses held. For what purpose? ask some one else. There was a strong under-tow of excitement, engendered by some personal differences, which, it is hoped, are now at an end. "Peace," "harmony," seemed to be the watch-words, and the *one thing* desired by those who may be said to be the leaders, —peace upon almost any terms. The final action of the Association would seem to demonstrate that for the sake of peace and cohesion, its policy—inconsistent tho' it may be with its record, —is not to look too closely into everything; better to shut eyes to some things than enforce too strict a construction of the Code, and strain the ship's timbers, perhaps. But are all the elements of discord eliminated, and will the peace be permanent? (The majority have made a big sacrifice to get it.) Is peace so sweet or harmony so dear as to be purchased at . . . . the price paid for it? Would not even shipwreck and a fresh start be preferable to the record just made and the present status?

The interpretation put upon it is, that the JOURNAL'S standard, to which an unvarying conformity has ever been insisted upon, was considered not practicable, at present; or, at least, that its enforcement was inconsistent with the corporeal integrity of the Association. There were some things, however, they could not stand; tho' all must recognize the premature calling of a halt, and stopping short of a clearly defined duty; and, construe it as one may, the majority has surrendered to a captious minority. They, the majority, have developed an unlooked for weakness in the spinal column; have sacrificed consistency for a questionable expediency, and go on record as *respecters* of *persons* rather than of principles. And the question, like Banquo's ghost, *will* arise; *have* the best interests of the Association been subserved?

The newly elected officers are representative men of the highest element. Dr. Wilkes, the President elect, is a popular favorite, whose flow of language is as voluminous and as sparkling as is the gush of their giant geyser.

Dr. Coleman, the First Vice-President, is a representative of the younger element of the most advanced workers and thinkers; and in honoring him, the Association honors itself and those he represents. Dr. West, the new Secretary, is Professor of Practice, and Dean of the Texas Medical College at Galveston. As a physician his ability is well known. No doubt he will make an excellent Secretary and Chairman of the Publishing Committee.

Waco is a delightful place, and her citizens are delightful people. They vied with each other in doing honor to their guests, and entertained them in a most charming and agreeable manner. The receptions given by the *elite* in honor of the Doctors were as brilliant as they were cordial, and all was done that could be done to make their guests welcome. It was a most enjoyable occasion, and will live long in the memory of those who had the good fortune to be present.

The details of the business transacted will be found in this number of the JOURNAL.

The exhibition of instruments and appliances and of pharmaceuticals, was grand; it constituted one of the most interesting features of the occasion. Few who have not attended such meetings recently, can form any idea of what perfection has been reached in the preparation of medicine for convenience of administration, and of the elegance of such preparations; nor of the advancement made in surgical instruments and appliances. Everything a doctor or a surgeon or an accoucheur could possibly need in an emergency is prepared ready to his hand. Instruments for precision in diagnosis, and for every phase of surgery, have been brought to great perfection. Many of the largest manufactories were represented by courteous agents; and to mention one, without naming all, would be thought hardly fair; but we never saw such a display of instruments as that of Mellier Drug Company—in charge of Mr. Albans.

The Association will meet at Tyler—the "Cradle of Judges" next April—4th Tuesday. May all honorable and good members live to be present. Texas medicine is no longer "an unknown quantity."

